# Assessing Tetraplegic Patients’ Neuro-Muscular Adaptations to a Six-Week Physiotherapeutic Programme

**DOI:** 10.5539/gjhs.v4n5p30

**Published:** 2012-07-05

**Authors:** Kayode Israel Oke, Oluwaseun S. Kubeyinje, Elias O. Agwubike

**Affiliations:** 1 Department of Physiotherapy, University of Benin Teaching Hospital, Benin City, Nigeria; 2 Department of Health, Environmental Education and Human Kinetics, University of Benin Teaching Hospital, Benin City, Nigeria

**Keywords:** complication, mobilization, physiotherapy, prevention, tetraplegics

## Abstract

Spinal cord injury is a life-transforming condition of sudden onset that can have devastating consequences. A multidisciplinary, functional goal-oriented programme is required to enable the tetraplegic patient live as fully and independently life as possible. Physiotherapy is a very important part of the multidisciplinary team required to prevent many of the immobilization complications that may result in serious functional limitations, reduce overall morbidity and achieve well patterned recovery. This study therefore highlights the neuromuscular adaptations of tetraplegic patients to physiotherapy over a period of six weeks. Fifteen patients participated in this study and the results showed that even though changes in the musculoskeletal parameters are inevitable in tetraplegics, the extent/degree of reduction of these parameters was grossly minimized in the studied subjects through the administration of physiotherapeutic measures. However, further research using a large sample size will be required to evaluate the physiologic adaptations of the neuromuscular system to the physiotherapy interventions among patients with spinal cord injury.

## 1. Introduction

Spinal cord injury (SCI) is a life transforming condition with a great risk of development of an array of debilitating and potentially life- threatening complications (Beilby & Mulligan, 2008). Its clinical management involves trauma management in the acute phase and rehabilitation which is lifelong.

Tetraplegic patients have impairment of motor and/or sensory function in the cervical segment of the spinal cord (Rowley, Forde, Glickman, & Middleton, 2001). Tetraplegia is a preferred term to quadriplegia and it presents with loss of muscle strength in all four extremities, the trunk and pelvic organs. Spinal cord injury damages a complex neural network involved in transmitting, modifying and coordinating motor, sensory and autonomic control of organ systems (Rowley et al., 2001).

The post traumatic dysfunction of the spinal cord causes loss of homoestatic and adaptive mechanisms which keep people naturally healthy. There are diverse causes of SCI but any mechanism that causes injury to the vertebral column or the bones in the back can damage the spinal cord. These causes can be traumatic (84%) or non-traumatic (16%) (Rowley et al., 2001). The most common causes are either motor vehicle accidents where the bones are broken or spinal joints are either partially or completely dislocated or gunshot injuries where the bullet damages the spinal column and the spinal cord (Solagberu, 2002). The authors’ personal observations and experience in their working places have witnessed a variety of such cases that collaborate with this situation. Other common causes include stabs, falls from heights, and diving accidents. Non-traumatic causes of spinal cord injury include tumors affecting the bones and then causing pressure on the nerve structure or the spinal cord itself. Other possible causes are a hernia at the disc that sometimes causes pressure on the spinal cord. There is also vascular cause in which the blood supply to the cord is damaged be that from an injury to the blood supply during surgery or other traumatic causes of the disruption of the blood supply which then disrupt the function of the spinal cord (www.spinal-injury.net).

Traumatic spinal cord injury (SCI) in the UK affects an estimated 10–15 people per million population per year so there are around 40,000 individuals in the UK living with a traumatic SCI (Gall & Turner-Stoke, 2008). The estimated population of SCI in the United States is 200,000, with the addition of 7,000 new cases each year (Elokda, Nielsen, & Shields, 2000; French et al., 2007), in which most injuries are in young men but the mean age of injury is increasing, including those injured over the age of 60 years (Santiago, Coyle & Kinney, 1993). More than one half of all cases of SCIs (56.4%) occur at the cervical level (Zimmer, Nantwi & Goshgarian, 2007). Male to female ratio is believed to be 5:1, though this varies with age as well as from country to country (Mitra, 2009). It has been reported that an average of 50 cases per year are admitted to a tertiary health institution in Benin City, Edo State, Nigeria, as spinal cord injured patients with the age ranging between 21-40 years (Udoh, 2008). It is believed to occur less commonly in ages less than 10 and ages greater than 60 with a ratio of males to females as 4:1 (Rowley et al., 2001). The majority of injuries that result in tetraplegia are predominantly incomplete injuries (Gall & Turner-Stoke, 2008). The prevalence of other conditions causing SCI such as inflammatory, neoplastic and infective conditions is currently unknown (Gall & Turner-Stoke, 2008). Traumatic SCI causes extensive human suffering and costs the society between 4 and 7 billion dollars annually (Stover, Delisa, & Whiteneck, 1995; French et al., 2007). Much of the cost can be attributed to secondary complications associated with degeneration of the musculoskeletal system of the paralysed extremities (Mitra, 2009).

Bielby and Mulligan (2008) outlined the following sequelae of tetraplegia secondary to SCI:

Joint contractures or permanent limitations of joint movement usually due to poor positioning, lack of movement and/or muscle spasticity; muscle atrophy, a shrinking or wasting of musculature due to lack of use; osteoporosis, deterioration of the bone that may occur due to decreased weight bearing, as well as factors related to the injury itself; development of deep venous thrombosis and possible pulmonary embolism due to lack of or reduced muscular pump activities for adequate venous return resulting from paralysis or weakness of muscles; and decreased respiratory function which can create such problems as increased risk for respiratory infections, congestion, rapid breathing and/or increased shortness of breath.

Management of the tetraplegic patients requires a multidisciplinary team approach, physiotherapy being one of the major inputs required in the care. Physiotherapy commences as soon as the patient is admitted and he/ she is stable clinically. The main objectives of physiotherapy in the acute phase include prevention of the above listed risks, maintenance of full range of motion (ROM) of all joints within the limitations of the injury, maintenance/ strengthening of all innervated muscle groups and to monitor neurological status and manage appropriately (Rowley et al., 2001).

Comprehensive rehabilitation (physiotherapy as a key part) along with early surgery, where necessary, is believed to markedly reduce the overall morbidity of spinal cord injured patients by enabling the patient to lead an independent life (Mitra, 2009).

Physiotherapeutic inputs have been considered an important adjunct in the care of the tetraplegic patients following spinal cord injury but no study has systematically and subjectively documented to what extent these measures or inputs maintain and improve functions towards eventual mobilization of tetraplegics after the acute care. This study therefore aims to fill this gap in knowledge.

### 1.1 Research Questions

The following research questions were raised to guide the study.

Would physiotherapeutic inputs achieve the expected goals in the recovery pattern / trend of tetraplegic patients? Specifically, the investigation probed into whether there would be a maintenance or improvements in the muscle strength, muscle girth, and range of movement among tetraplegic patients following physiotherapy measures in the acute phase of their management?

### 1.2 Hypotheses

The following hypotheses were formulated and tested.

Physiotherapeutic inputs would not significantly contribute positively towards mobilization of spinal cord injured patients with tetraplegia during the acute phase of their management.

The specific hypothesis formulated and tested was whether there would be no significant improvements in the muscle strength, muscle girth, and range of movement among tetraplegic patients following physiotherapy measures in the acute phase of their management.

## 2. Methods and Materials

### 2.1 Subjects

Fifteen subjects participated in this study which comprised of thirteen (13) males and two (2) females with average ages of 29.3 and 37.0 years old respectively. They were randomly selected from those admitted in the neurosurgical ward of the University of Benin Teaching Hospital, Benin City, Nigeria, between 2009 and 2011. The age range of the males was between 12 and 50years old while that of the females was between 27 and 47 years old.

Inclusion criteria was the enrollment of patients with quadriplegia resulting from SCI (arising from trauma to the cervical spine) without associated injuries like head injuries, psychiatry condition, fractures of the limbs or ribs; medical conditions like diabetes mellitus and positive retroviral states. Exclusion criteria included aged patients of over 65years old and those with chronic (old) SCI. Ethical approval of the research and ethics committee of the study institution was sought and obtained.

### 2.2 Measurement Procedure

This study measured musculoskeletal parameters of fifteen (15) tetraplegic patients at the commencement of physiotherapy immediately at their being admitted to the neurosurgical ward, two weeks, four weeks and six weeks post admission. The progress or otherwise on the specified variables during the period of immobilization were compared. Physical measurements of these parameters were taken using validated instruments at the different stages of care.

The present physiotherapeutic measures employed in the management of SCI patients with tetraplegia were Chest Physiotherapy, Passive Mobilization, Proper Positioning on bed, strengthening exercises, soft tissue mobilization, pain management procedures and preparation for mobilization.

Parameters that were measured adapted Adams and Hamblen’s (1990) protocol which focused on muscle strength, muscle girth, range of joint movements/motion, forced expiratory volume and maximum inspiratory volume/capacity. Muscle strengths were measured subjectively using Medical Research Council grading as follows: 0= no contraction; 1= a flicker of contraction; 2= slight power, sufficient to move the joint only with gravity eliminated; 3= power sufficient to move the joint against gravity; 4= power to move the joint against gravity plus added resistance; 5= normal power (Adams & Hamblen, 1990).

Muscle girths were measured using measuring tape rules. The girths of arms, forearms, thighs and legs were measured in these patients using the International Standards for Anthropometric Assessment (ISAK) recommended points and positions. The bilateral arm girth measurements were taken at the level of the mid acromiale-radiale with the patients assuming a relaxed supine lying position and the elbow being in an extension with the tape positioned perpendicular to the long axis of the arm.

The forearm girths bilaterally were taken at the maximum girth of the forearm distal to the humeral epicondyles with the forearm supinated while relaxing the muscles of the forearm. For consistency in the measurement, a point 10cm away from the tip of the olecranon was marked as the reference point. For the thigh and leg girths measurements, a distance of 12.5cm above and below the upper and lower tips of the patellae was used as the reference point for the purpose of uniformity. The patients were measured in a relaxed supine lying position with the knee joints extended. In all the girth measurements, it was ensured that the tape was firmly passed round the limbs without excessive tension and that it did not indent the skin or slip. Three measurements were taken on each point and the average values of the measurements were taken and recorded.

The joints range of motion of all the patients were measured with the aid of a goniometer. The joints used in this study were the shoulders, elbows, wrists, hips, knees and ankle joints bilaterally. Measurements were also taken at two, four and six weeks on admission.

The neurosurgical management of a tetraplegic compulsorily requires a period of six weeks of immobilization on bed for postural reduction. Tetraplegic patients admitted in the neurosurgical ward in supine lying positions with either a cervical collar (rigid collar with jaw extension, or Philadelphia collars) or Crutchfield tongs/calipers or Gardner-Wells traction fixed on the skull depending on the degree of instability to immobilize the injured cervical spine for spinal stabilization were immobilized on bed for an average of six weeks. A few of the patients with unstable spine or observed not to have improved satisfactorily with conservative approach were operated upon using surgical procedures for spinal stabilization. In such an instance, they spent less than six weeks on bed post operation for proper spinal stabilization and rehabilitative care. All the patients had daily physiotherapy during the acute phase of their management. The daily treatment consisted of manual therapy of Chest Physiotherapy of vibration and shaking of the chest for about 5- 10minutes along with active deep inspiration exercise. Passive mobilization of all the extremities of the patients carried through safe range or degree, strengthening exercises all the muscles of the extremities, soft tissue mobilization with aid of talcum powder, proper positioning of the limbs on bed, pain management procedures and preparation for mobilization in the form of ambulation when due. The treatment regimen takes an average of one hour per patient daily and each patient had four treatment sessions per week. The treatment period was six week of postural reduction on bed. The data obtained were fed into SPSS version 16 statistical analysis. Descriptive statistics of mean, standard deviation and percentage as well as inferential statistics of one-way repeated measures ANOVA were used to test the hypotheses at alpha level of 0.05.

## 3. Results

The results are presented in tables 1-12 as contained below.

**Table 1:** Chest and waist circumferential measurement

Circumferential Measurement	1^st^ (Mean±SD)	P-value for 1^st^ & 2^nd^ CM	2nd(Mean±SD)	P-value for 1^st^ & 3^rd^ CM	3rd (Mean±SD)
Waist circumference	72.18cm±10.92	0.000	71.49cm±10.24	0.000	77.25cm±10.46
Chest circumference	77.86cm±9.63	0.210	75.31cm±9.53	0.001	78.25cm±9.58

SD= Standard Deviation. CM= Circumferential measurement

**Table 2:** Patients’ muscle tone improvements

Measurement	Right		Left	
	Upper Limb	Lower Limb	Upper Limb	Lower Limb
1^st^ Assessment	H	H	H	H
2^nd^ Assessment	H	H	H	H
3^rd^ Assessment	H	H	H	H

H = Hypotonic

**Table 3:** Muscle girth measurements (cm)

Regions Measured			1^st^ Measurement	2^nd^ Measurement	3^rd^ Measurement
Right	Upper	AE	24.16± 4.74	22.78± 4.94	23.10± 2.88
	Limb	BE	18.23± 2.11	19.16± 4.23	19.70± 3.38
	Lower	AK	40.74± 2.50	37.85± 3.41	39.93± 2.63
	Limb	BK	29.55± 4.84	27.82± 5.11	27.55± 4.63
Left	Upper	AE	23.84± 3.84	22.82± 3.94	23.23± 3.78
	Limb	BE	19.41± 3.36	19.54± 5.24	18.90± 3.06
	Lower	AK	38.88± 2.68	37.68± 2.85	37.35± 5.71
	Limb	BK	28.33± 2.43	27.33± 3.11	27.70± 3.71

AE= Above elbow; BE= Below elbow; AK= Above knee; BK= Below knee

**Table 4:** Range of motion improvements

JOINTS	1^st^ Assessment	2^nd^ Assessment	3^rd^ Assessment
Shoulder	0° - 90°	0° - 90°	0° - 90°
Elbow	FR	FR	FR
Hip	FR	FR	FR
Ankle	FR	FR	FR

FR = Full Range

**Table 5:** One-way ANOVA results of the body regions measurements

		Sum of Squares	Df	Mean Square	F	Sig.
Right Upper Limb (Above Elbow) Second	Between Groups	308.234	12	25.686	1.522	.464
	Within Groups	33.760	2	16.880		
	Total	341.994	14			
Right Upper Limb (Above Elbow) Third	Between Groups	116.185	12	9.682	77.457	.013*
	Within Groups	.250	2	.125		
	Total	116.435	14			
Right Lower Limb (Above Knee) Second	Between Groups	161.634	12	13.469	19.151	.051*
	Within Groups	1.407	2	.703		
	Total	163.041	14			
Right Lower Limb (Above Knee) Third	Between Groups	95.417	12	7.951	9.898	.095
	Within Groups	1.607	2	.803		
	Total	97.024	14			
Left Upper Limb (Above Elbow) Second	Between Groups	216.111	11	19.646	235.757	.000*
	Within Groups	.250	3	.083		
	Total	216.361	14			
Left Upper Limb (Above Elbow) Third	Between Groups	164.047	11	14.913	1.257	.478
	Within Groups	35.585	3	11.862		
	Total	199.632	14			
Left Lower Limb (Above Knee) Third	Between Groups	450.351	12	37.529	11.161	.085
	Within Groups	6.725	2	3.363		
	Total	457.076	14			
Left Lower Limb (Above Knee) Second	Between Groups	113.330	12	9.444	59.026	.017*
	Within Groups	.320	2	.160		
	Total	113.650	14			
Right Upper Limb (Below Elbow) Second	Between Groups	247.803	12	20.650	16.783	.058
	Within Groups	2.461	2	1.230		
	Total	250.264	14			
Right Upper Limb (Below Elbow) Third	Between Groups	156.308	12	13.026	7.647	.121
	Within Groups	3.407	2	1.703		
	Total	159.715	14			
Right Lower Limb (Below Knee) Second	Between Groups	365.985	13	28.153	1.408E3	.021*
	Within Groups	.020	1	.020		
	Total	366.005	14			
Right Lower Limb (Below Knee) Third	Between Groups	297.153	13	22.858	7.031	.288
	Within Groups	3.251	1	3.251		
	Total	300.404	14			
Left Upper Limb (Below Elbow) Second	Between Groups	384.413	13	29.570	92.407	.081
	Within Groups	.320	1	.320		
	Total	384.733	14			
Left Upper Limb (Below Elbow) Third	Between Groups	129.141	13	9.934	4.967	.339
	Within Groups	2.000	1	2.000		
	Total	131.141	14			
Left Lower Limb (Below Knee) Second	Between Groups	122.880	12	10.240	1.608	.447
	Within Groups	12.740	2	6.370		
	Total	135.620	14			
Left Lower Limb (Below Knee) Third	Between Groups	137.713	12	11.476	.416	.867
	Within Groups	55.127	2	27.563		

* Significant @ 0.05 level

Table 1 presents the waist and chest circumferential measurements for all the patients. The initial measurement of 72.18cm only reduced by 0.95% after two weeks of immobilization and later increased by 7% after another two weeks of immobilization with daily deep breathing and incentive spirometric exercises. All the waist girth circumferential measurements were taken one hour postprandial. Trunk circumference measurements were also represented in Table 1. The trunk circumference measurement was used to determine the extent to which the lower abdominal muscles stretched or contracted to compress the abdominal cavity in pushing the diaphragm up to push air out of the lung. The initial mean value (1st measurement) was 77.86cm, which reduced by 3.3% to 75.31cm after two weeks on admission and later increased by 0.5% to 78.25cm after another two weeks.

Table 2 presents the muscle tone of all the patients. The tone was reduced throughout the period by retaining its hypotonic status. Table 3 shows the tabular representation of the values of muscle girths measured on the patients along with the regions of the body where the measurements were taken. The initial mean value of the right upper limb at a point 12.5cm above the elbow was 24.16cm. It reduced by only 5.7% (22.78cm) after two weeks and there was a gain of 1.3% (23.10cm) on the second assessment when the third measurement was taken on the sixth week. The initial mean value of the knee measurements of the left lower limbs taken at a point 12.5cm above the superior part of the patella was 38.88cm. It became 37.68cm (which represented only 3.1% reduction in limb girth mass) after two weeks of daily passive mobilization and became 37.35cm (which was 3.9% reduction from the initial lower limb mass at 12.5cm above the superior part of the patella) in the last two weeks. Values of the measurements of the other regions of both upper and lower limbs as measured initially, two weeks and four weeks later are contained in Table 3.

Table 4 presents the range of motions (ROM) on the shoulder, elbow, hip and ankle joints of the patients as observed within the period of the study. It showed that the ROMs maintained the initial measured ranges through daily passive range of motion exercises. The normal ROM used for the shoulder joint in this study was 0 - 90degrees because of the fact that the shoulder joints of tetraplegic patients on cervical spine stabilization procedures, especially those on Gardner Wells traction, were not advised to be moved through full range during the acute care phase. The range of motion of the knee joint was intentionally excluded in this study because of the chances of development of deep venous thrombosis (DVT) in the lower limb(s) of the patients which may lead to stiffness of the knee joint due eventually to withdrawal of passive mobilization of the knee joint in event of DVT.

In terms of inferential statistics using one-way repeated measures, three out of sixteen parameters were not statistically significant. Specifically, Table 5 shows that the right upper limb, above elbow, for second measurement was statistically significant (p= 1.522 with 0.464 significance) while the third measurement (p= 77.457 with 0.013 significance) was not significant.

In Table 5, the values of 19.151 with 0.051 and 9.898 with 0.095 for right lower limb (above knee) second and third measurements were all statistically significant. Table 5 also shows that significant values were obtained for the left upper limb (above elbow) for second and third measurements (p= 235.757 with 0.000 and 1.257 with 0.478). However, for left lower limb (above knee), the third measurement in Table 5 indicated a statistically significant value (p= 11.161 with 0.085) while the left lower limb (above knee) showed a non-significant value (p= 59.026 with 0.017) for second measurement.

The second and third measurements for right upper limb, below elbow, showed statistically significant values of 16.783 with 0.058 and 7.647 with 0.058 respectively as shown in Table 5. The table also shows non-statistically significant value for right lower limb (below knee) in second measurement only (p= 1.408 with 0.021 while the third measurement shows a statistically significant value (p= 7.031 with 0.288). Table 11 and 12 for left upper limbs for below elbow and knee showed statistically significant values for both the second and third measurements (p= 92.407 with 0.081 and 4.967 with 0.339 as well as 1.608 with 0.447 and 0.416 with 0.867).

## 4. Discussion

The purpose of this study was to investigate effect of early physiotherapy measures on musculoskeletal parameters (muscle strength and girth, and joint range of movement) that are required for functional activities in patients with spinal cord injury (SCI).

The outcome of the study revealed significant differences in the muscle girth measurements in majority of the body segments of the extremities of the patients measured. The study revealed no significant difference in the reduction of the chest circumferential measurements taken two weeks on admission but a significant difference in the increase in the circumferential measurement obtained by six weeks during rehabilitation. However, there were significant differences both in the initial reduction and the eventual increase in the waist circumferential measurement of the patients during the six weeks period of admission for postural reduction. The chest and waist circumferential measurements were used as surrogates for chest and abdominal muscle strength and lung ventilation capacity (inspiratory and expiratory functions) in this study. Respiration is a function that is critical for sustaining life but also significantly important for generating the pressure needed to cough, speak, and swallow (Kim & Sapienza, 2005). Respiratory insufficiency (which often results from paralysis and/ or weakness of respiratory muscles) is the number one cause of mortality and morbidity after SCI (Zimmer, Nantwi & Goshgarian, 2007). The outcome of this study is in agreement with the observation of Silveira, Gadstaldi, Boaventura and Souza (2010) that exercise training can improve inspiratory muscle strength, forced vital capacity (FVC) and expiratory muscle performance in quadriplegic patients. Kim and Sapienza (2005) reported that loss of strength and mass of lateral internal intercostal and abdominal muscles (which are main expiratory muscles) can result in deterioration of both ventilatory and nonventilatory functions. The study showed significant differences in muscle girth measurements in majority of the regions measured on the upper and lower extremities of the participants with insignificant differences in changes in the girth of some regions of the upper and lower extremities muscles. The insignificant changes were majorly observed in the measurements taken within the second and third measurements interfaces, that is, towards the end of postural reduction. The observed initial reductions in muscle girth in these patients were in agreement with previous observations of other researchers (Castro, Apple Jr, Hillegass, & Dudley, 1999; Giangregorio & McCarteney, 2006). Research reports have indicated that after SCI, there is a rapid and dramatic loss of muscle mass below the level of the lesion (Castro et al., 1999; Giangregorio & McCarteney, 2006). During the first months postinjury, demineralization occurs exclusively in the sublesional areas and predominantly in weight-bearing skeletal sites such as the distal femur and proximal tibia, which are trabecular-rich sites, while the diaphyseal areas of the femur and the tibia, which are cortical-rich sites, are relatively spared (Maïmoun, Fattal, Micallef, Peruchon, & Rabischong, 2006). Giangregorio and McCarttney (2006) have reported between 16- 46% reduction in lower extremities muscle mass within 6 weeks post SCI in affected individuals. However, the reduction in girth of most of the regions measured in this study was between 4.4-8.1% (mean= 4.2%), and therefore did not experience such a large difference. The intervention of regular passive mobilization, assisted active strengthening exercises and soft tissue mobilization techniques where necessary may have helped in reducing the rate and volume of muscle girth losses in these patients which is also thought to have resulted in differences that were not significant on inferential statistics in some of the body regions especially in the upper extremities. The tone of the muscles of the participants remained hypotonic during the studied period. Hypotonia is one of the primary motor symptoms in acute stage of spinal cord injury (Mitra, 2009). Hypotonia may however change to hypertonia during rehabilitation phase. Regular turning and positioning in bed were used to minimize spasticity and to control increased flexor tone, muscle imbalance and soft tissue shortening that could result in pain (Rowley, Forde, Glickman, & Middleton, 2001).

Turning and positioning are measures that can minimize spasticity and prevent flexor muscle hypertonia in SCI patients who are recumbent on bed. The study also recorded full range of motion on all the joints of the extremities measured throughout the study period. This has been achievable through the daily administration of passive mobilization of the limbs by the physiotherapists. Contractures (reduced joint mobility) were due to loss of extensibility in soft tissues spanning joints, and were found to be a common complication of spinal cord injury. One study found that spinal cord injured patients had, on average, seven contractures at between 6 and 7 weeks after injury (Harvey & Herbert, 2002). Contractures were undesirable for many reasons but primarily because they prevented the performance of motor tasks. For example, elbow flexion contractures made it difficult for tetraplegics with paralysis of triceps muscles to bear weight through the upper limbs, and hence could attain independence with transfers. Harvey and Herbert (2002) observed that contractures also created unsightly deformities and were thought to predispose patients to spasticity, pressure sores, sleep disturbances and pain. The present study however showed that regular physiotherapy in the acute phase of care successfully reduced the risk of developing contractures to the barest minimum. Proper positioning and frequent turning on bed were effected as an essential part of the acute phase management of the tetraplegic. The extent to which a tetraplegic patient was able to function after recovery especially with the limbs was highly dependent on the outcome of the acute phase care. Proper positioning on bed prevented complications like pressure ulcers which are among the most costly complications of immobility in terms of added morbidity and cost. Its prevalence in the acute care setting is estimated to be between 3% and 28%; with an incidence of 1% to 8% during hospitalization (Goode & Allman, 1989). It has been estimated that pressure ulcers can account for approximately one-fourth of the cost of care for individuals with spinal cord injury (Bogie, Reger, Levine, & Sahgal, 2000). Patients with insensate skin, physical impairments, inability to perform pressure relief like some tetraplegic patients, were at even greater risk for the development of pressure sore (Allman, Laprade, Noel, Walker, Moorer, Dear, & Smith, 1986; Reddy, Gills, & Rochon, 2006). The present study reduced the risks of these conditions degenerating into serious complications. Upper limb positioning was properly executed in the tetraplegic patient as a very important care strategy during the acute phase of care for the tetraplegic patients. Positioning was used to minimize spasticity in the patients and to control increased flexor tone, muscle imbalance and soft tissues shortening that could result to pain as revealed by Rowley et al (2001). Muscles that were immobilized and remained in a shortened position or fixed position became contracted and eventually, much of the muscle tissue was replaced by fibrous components and normal function could not be restored (Stewart, 1989). The physiotherapists also carried out the positioning with the anatomical muscle origination, insertion and action in mind so as to prevent oedema in distal parts of the limbs like the wrists and feet. Management of the tetraplegics in the acute phase required the use of splint in achieving correct positioning. A splint that immobilised the metacarpophalangeal (MCP) joints in flexion and interphalangeal (IP) joints in extension helped to prevent MCP hyperextension and IP flexion contractures both of which are common in tetraplegics with lesions at or above C5, particularly if oedema is also present as recommended by Harvey and Herbert (2002). Strengthening exercises were carried out to assist muscles that were recovering from motor functions to regain functional muscle powers. It is a known fact that pain increases susceptibility to contracture because it increases the tendency to contract non-paralysed muscles, which in turn, increases the time soft tissues spend in shortened positions. Occurrence of pain in spinal cord injured patients is associated with limitation of function and poor pattern of recovery, though the extent to which pain affects these is still a subject of debate. In the present study, it was registered that pain was not usually a problem in the acute phase of management except if there were associated musculoskeletal injuries like ribs and limbs fractures. Patients with such kinds of associated injuries were excluded from the present study. It was observed that pain especially joint pains in tetraplegics were usually as a result of development of soft tissues stiffness and contractures which occurred when preventive measures like proper positionings, regular joint mobilization exercises were not carried out. Such pains were grossly reduced in the present patients probably because proper positioning and regular joint mobilization were effectively and efficiently executed. Such pains have however been linked with times or duration of injuries by little available researches on pain in SCI injured patients.

Jensen, Hoffman and Cardenas (2005) revealed that in one retrospective survey, very few respondents indicated that pain had decreased in intensity (6.6%) or frequency (7.1%), while many more indicated increases in both intensity and frequency since the onset of their SCI (47.2 and 33.5%, respectively). Another study found that time since injury predicted the prevalence of shoulder pain in a sample of men with SCI, with greater time since injury associated with a greater frequency of shoulder pain, and one study found that time since injury was associated with a higher prevalence of elbow and shoulder pain, but not with ankle pain or pain in general (Jensen, Hoffman, & Cardenas, 2005). Also, the presence of pain showed a trend to be associated with poorer psychological functioning and social integration, and the intensity of pain was associated with interference with a number of important basic activities of daily living (Jensen et al., 2005).

In the present study, occurrence of pain was anticipated and was also prevented by the use of medications by the neurosurgeon to avert occurrence of chronic pains. Topical analgesic gels were also applied on the joints of the upper and lower extremities to relieve pain where applicable. These were gently massaged into the joints by the physiotherapists; patients’ relatives were taught how to apply these gels where necessary in order to ensure twice or thrice daily applications when the severity of pain demanded such.

## 5. Conclusion and Recommendations

This study has presented results that further strengthened the effects of physiotherapeutic measures as important adjuncts in the management of SCI patients with tetraplegia especially during the acute care phase of management. It showed that even though changes in the musculoskeletal parameters are inevitable in tetraplegics, the extent/degree of reduction of these parameters can be reduced grossly through the administration of physiotherapeutic measures. It was evident from the results that the rate of neurophysiological responses that follow the spinal cord injuries which result in deterioration of neuromuscular parameters like the muscle mass, tones, power and joint flexibility can be greatly influenced by application of physiotherapy techniques and measures. Therefore, it is highly recommended that structured physiotherapeutic measures should be an adjunct to the care and management of SCI patients. It is also recommended that further studies that will make use of larger samples and that will also follow up effects of physiotherapeutic measures on recovery pattern of patients with SCI beyond the acute stage be conducted.
